# Reviews

**Published:** 1884-04

**Authors:** 


### REVIEWS.

The Relation of Animal Diseases to the Public Health, and their Prevention, with a Brief Historical Sketch of the Development of Veterinary Medicine, from the Earliest Ages to the Present Time; and a Critical Historical Sketch of the Leading Schools of the World, showing the Reasons which led to their Foundation, and with the Endeavor to draw from their Experiences Teachings of Value toward the Establishment of a General Veterinary Police-hygienic System and Veterinary Schools in this Country. By Frank S. Billings, Veterinary Surgeon, Graduate of the Royal Veterinary Institute, Berlin; Member of the Royal Veterinary Association of the Province of Brandenburg, Prussia; Honorary Member of the Veterinary Society of Montreal, Canada, etc., etc. 1 vol., 8vo, pp. 440. D. Appleton & Co., Publishers, New York.

This handsome volume does great credit to its author and publishers. The preface says it is "written for the benefit of the people of the United States. Its purpose is to introduce to every thinking man and woman of the country a new subject, viz: the higher purposes of veterinary medicine. It is a work which treats of the *Prevention* of Diseases, not their *Treatment.* While at times the language of the author may appear unnecessarily severe to the casual reader, he should not forget that the author is an enthusiast; that he has given his life and energies to the subject of the establishment of veterinary science in this country; and that the evils so severely combatted are not the creations of a vivid imagination, but actual evils, that unless prevented, will work most serious injury to the country in the not distant future. All that the author asks is calm reflection and an honest verdict upon his work."

It is rather difficult for all, except the most Christian, amiable, or wisest people to treat very aggressive ones with that "calm reflection," which is most requisite for a just verdict upon their work. But a careful second reading of the book robs most of these blemishes of their terrors. It is an excellent book in most respects; an extraordinary one in many, and an objectionable one in very few. It at the very least should be in the libraries of every National, State, city, town, and County Board of Health. It certainly should be studied by every teacher and scientific practitioner of veterinary medicine, and will be of great service to every great stock and cattle holder and dealer.

Some even thinking men and women may be frightened off by the occasional use of unnecessarily hard words and phrases, but most of the book is very readable and interesting indeed, and exceedingly instructive. It is evidently written by a man of great ability and high culture, well versed, both in the literature and science, as well as the practical bearings of his subject. Such a man has a great and inalienable right to have opinions of his own ; and he has them, and does not hesitate to express them.

Although the work is a well connected and compact whole, it still consists of two great parts, almost like two distinct volumes.

Part 2d, containing the history of Veterinary Medicine, and reaching from page 209 to 241, will be welcomed by all good scholars, especially those, and their name is legion, who own or love animals. It should be well conned by all Health authorities, and the first and prompt effect of this study should be the appointment of a capable veterinary doctor and surgeon in each and every Board of Health, tending to the better hygienic care of all domestic animals, and the speedy recognition of their infectious and contagious diseases, and the quick stamping of them out by competent men having full authority.

The author was mainly educated in Germany, notably in Berlin, Vienna, and Munich, and of course we have veterinary medicine and its history mostly from the German standpoint, which is admitted to be the most scientific. But he is almost as much at home with the French language, and the history of French Veterinary Medicine is nearly as complete as the German. Both will be read with great interest and profit. British and American history is more lightly touched upon, but that is perhaps reasonably well known to all reading veterinary men. The principal lesson to be learned from all this history is, that all concerned in the care of animals, down to cowherds, shepherds and farriers, should have some good knowledge of the anatomy and physiology of animals. Even dentists now study medicine at large, and scientific veterinary medicine is now making such great strides that the humblest attachés should at least have the inklings of what is expected of "trained nurses." They may not be able to read this book with ease and pleasure, but their masters and every one who claims to be a good veterinary doctor should.

The first part, pages 1 to 208, is taken up with the consideration of some of the most important infectious and contagious diseases of animals: those which require both scientific knowledge and official authority to examine and repress.

"Trichiniasis" in men and animals is dealt with in pages 1 to 40, and is a capital study indeed. The ready detection of the disease in slaughtered hogs, about the pillars of the diaphragm, is especially important. But the autnor has Bismarckian views about the "great American hog," which may raise an unjust howl from those whose pockets will be touched. We hope they will, for the intrepid doctor is fully capable of dealing with them, and he should have his chance. It will be a hard fight and a good one. Before government acts in the matter, all large pork packers should have skilled examiners, licensed from some good veterinary college, to inspect and mark their products. These will find a more ready sale at higher prices than less well attested articles. These certificates will doubtless have a higher standing than those of some government officials, appointed for some political reasons only or mainly.

Next to hogs, *Trichinae* are apt to infest *rats*, and the doctor says: "Continued examinations of rats should be made in all parts of the country, and their slaughter encouraged in all legal ways. In this regard we can even look upon the rat pit as serving a useful public purpose, and the rat invasion theory, with reference to hogs, will receive a sooner final settlement." But Mr. Bergh will surely interfere here, and when Greek meets Greek, will come the tug of war. The directions for the prevention of Trichinae in swine, p. 31, are excellent, although little is said about disinfection, above which cleanliness, inspection, branding diseased hogs, etc., are preferred.

"Hog cholera" occupies pages 41 to 50. This chapter is short but excellent. The cause of the disease, Bacillus Suis, is well tracked down; the microscopical examinations well given, and the preventive measures thorough, down in extreme cases to slaughtering the infected animals in their own pens, and burning the latter with all contaminated wooden utensils. Sheep and rabbits are subject to what is called "hog cholera," and require attention in places where the disease prevails.

"Tape worm" in hogs and cattle is treated of in a short but masterly way. The *Taenia Medio-canellata* comes from beef, which is especially dangerous when eaten raw, or very rare. *Taenia Solium* comes from pork, and affects those who eat raw ham and under-done pork, and slightly smoked and cooked sausages. This chapter should have had a distinct heading, which is lacking, and may be overlooked by all who do not read the book regularly and carefully through. The same suggestion applies to the chapter on *Foot and Mouth Disease*, or *contagious eczema* of cattle. This infection is also apt to implicate sheep, swine, goats, deer, occasionally horses, and sometimes dogs and turkeys. Cases in children in New York have occurred apparently from the use of contaminated milk; and the disease is cropping up in various parts of the country, both far north and far west. It has possibly been imported by English cattle which have escaped quarantine inspection, although the spontaneous generation of a similar disease where cattle live in marshes and filth cannot well be denied. Eczema, or salt rheum, is the most common skin affection in human beings, and how much of it comes from cattle is not yet determined. Bollinger says: "Notwithstanding the ruling opinion to the contrary, the disease is much more common among human beings than is suspected." The suggestion of Dr. Billings that milk should be examined for much more than mere dilution with more or less pure water, is worthy of all consideration. This suggestion receives still greater emphasis in the chapter on "Tuberculosis in Cattle," pages 52 to 74, which is all too short, although pregnant with information. The credit of first calling attention to this dire disease is given to Gerlach, to whom Dr. Billings has dedicated his book. The notion that pulmonary consumption may be conveyed by the milk of tuberculosis in cows is not a pleasant one. In the opinion of the reviewer, consumption is often a foul air disease, caused quite as much and even oftener by inhaling foul air, as from mere exposure to cold and wet. Dr. B. says "in Germany where the majority of the milch cows are stall-fed, and that too in poorly-ventilated, ill-arranged and filthy stables, this disease has acquired an extension of which we can at present make no appreciation in this country;" although we have an inkling of it among swill-fed cows. Bollinger reproduced the disease in pigs, calves, lambs, and rabbits fed on milk from tuberculous cows. Billings is undoubtedly right when he says, page 73, "that such milk does contain elements of a specifically infectious character, and there is no question that laws should be made, and executed also, to prevent the sale of such milk for human consumption, either by itself, or mixed with other milk, in no matter how small quantities. No such milk should be sold. The specific infection of milk from tuberculous cows is no trifling matter; it is one of life and death." Consumption, scrofula and marasmus are only too common among the hundreds of thousands of babies that are yearly brought up on poor cow's milk. However important trichiniasis may be, this far exceeds it.

Every consumptive cow should be branded by expert men. Its milk can only be given with safety to swine after being boiled; and although the notion is not a nice one, the doctor thinks they should be fattened and killed, as the meat is not injurious when well cooked. It is to be presumed that even "the eaters of lights" will not consume the lungs of such animals, and the liver and kidneys also must be viewed with much suspicion.

"Anthrax, carbuncle, or malignant pustule" occupies a long chapter extending from page 71 to 124, including, however, section on infection or bacteria, which reaches from page 74 to 100. Although the Doctor does not suggest it, we venture to hint that the plague of boils among men may sometimes be traced to this source.

His views on bacteria are based upon those of Naegeli, although almost all good workers have been laid under contribution; and will well repay careful perusal, although they are not conclusive on some points which are well known.

The chapter on Disinfection, page 100, is especially unsatisfactory in the light of recent developments. Killing bacteria is now regarded as one of the great ends of preventive medicine. Fox and rabbit, deer and grizzly bear hunting sink into insignificance compared with it; and to be told that a special study of antiseptics would lead the Doctor too much into details for his present purpose, and that he must refer his readers to the special works upon the subject, is rather disappointing. Among the disinfectants, he says, are the corrosive acids, Carbol, Thymol, Boracic Acid, Permanganate of Potash, the coal tar preparations, and the long list of antiseptics." He rightly says: "We too often think when we have removed the odor from an offensive substance or place we have destroyed its disease-producing qualities, a hope which is only too soon negatived by further experience. The resistibility of germs is such that we may look upon it as very nearly fallacious to disinfect the atmosphere by adding to it chlorine gas, sulphur, or any similar disinfectants." And there he leaves us.

It is true that animals are such inconsiderate filth producing creatures that nothing will take the place of cleanliness; sweeping, washing, and drainage are all important, and no disinfectants, or even germ-killers, can take their place. Common bacteria cause putrefaction, and good and frequent lime-washing and sprinkling of lime, or better, unburnt gypsum, along the walks between and at the foot of the stalls, are not to be despised, however vigorously the broom and hose may be used. Carbolic acid is useful against common bacteria, or those which produce ordinary putrefaction and foul smells; it fails against the specific bacteria of diphtheria, hog cholera, anthrax, bovine tuberculosis, and other formidable pestilences. We know that quinine will kill the bacilli of fever and ague and that male fern will kill tape worms. But we do not know what will destroy trichinæ. We know that one form of bacteria and no other will cause erysipelas, and the specifics against this disease will kill them and prevent its spread to other patients.

Corrosive sublimate, bromine, iodine and turpentine are more efficient than carbolic acid, but they cannot always be used safely in the case of animals.

We hope in the second edition, which we trust will soon be called for, the Doctor will complete his chapter on this subject. The proper disinfection of horse-stables, which are so thickly scattered about in all towns and cities, is alone a subject which is fraught with the greatest benefit and comfort to many human beings who suffer from their vile smells.

The disinfection of cow-stables does not come home so directly and frequently to town-people, but advice to dairymen would not be out of place. Milk absorbs all odors and takes up many aerial germs. Counsel in this direction is all important, and the Doctor is perfectly competent to give it in the most efficient manner. Even if he gave us nothing especially novel, the stamp of his approval or disapproval would carry great weight.

The chapter on "Anthracoid diseases" is one of the most important. *Anthrax* means a *coal*, and physicians are apt to think of carbuncles and red hot coals at the same time. Veterinarians think of black and cool or cold coals and apply the term anthracoid, or anthrax-like, to a group of diseases characterized by a black, tarry appearance of the blood. Physicians think first of cattle plague, Steppe disease, Rinderpest, Maladie de Sang, Milzbrand, Splenic apoplexy, Charbon Braxy in sheep, etc., etc., and have been in the habit of comparing them to black typhus fever, or typhus contagiosus even down to the time of Parkes.

*Anthrax*, or malignant pustule, in man, apart from ordinary carbuncles, is caused by inoculation from anthrax-diseased animals or their products. Anthrax fever is caused by the consumption of the flesh of diseased animals. The infection in man, to the extent of 84 per cent. of all cases, occurs primarily on parts that are generally uncovered, such as the face, lower arm, hands and fingers, etc. Butchers, tanners, wool-pullers, and workers in horse-hair, are most subject to it; but flies and insects may also convey the disease to others. The principal anthracoid diseases, apart from Rinderpest, are: Emphysema contagiosum, which Parkes calls *Erysipelas contagiosum* or Black quarter, is the German's Rauschbrand.

Next comes the *Texas* or *Spanish cattle fever*.

All the polemics which the Doctor has been obliged to enter into, in properly clearing up other subjects, sink into insignificance compared to those which fall upon the opinions of Gamgee. The topic is so important to us, and is so well and powerfully handled, that all interested in cattle should read it. The Bacillus anthracis is well known, and another form occurs in Rauschbrand; but none as yet have been discovered in the Texas cattle disease or Splenic fever.

The next chapter on "Dog Diseases," page 137, deals with the diseases of dogs.

*Taenia echinoccus*, the smallest of tape-worms, which, however, leads in man and other animals to the formation of the hugest cysts or sacs, is sufficiently touched upon. Of 252 of these cysts, found in the human body, no less than 176 were in the liver, and 54 in the abdominal cavity, leaving only 25 for all other organs.

The exciting topic of "Rabies" in the dog occupies pages 139 to 153, and is replete with instruction. Like malignant pustule inoculation takes place most frequently in uncovered parts. Of 495 cases of hydrophobia in man,

263 or 53 per cent. came from bites on the hands and arms; 110 or 22 per cent. upon the head and face; and only 14 or 3 per cent. on the legs. Some of the other statistics are exceedingly interesting; thus only 8 persons died of hydrophobia out of 49 bitten by rabid dogs, 12 by suspected dogs, 74 by diseased dogs, 5,268 by healthy dogs, or 1 to 50.

The efficacy of prompt and good cauterization is shown by figures: Only 37 per cent. of the cauterized die, while 82 per cent. of those who neglect this succumb.

There were 23 mad cats to 707 mad dogs.

The disease, in a large proportion of cases, shows itself within the first sixty days after the bite. Thus, of 221 attacks 139 occurred within two months, 54 between 60 and 100 days, 21 between 100 and 180 days, and only 3 at later periods.

The consideration of the Horse and its diseases occupies from page 153 to 208. Of these to page 159 is taken up in the discussion on *Hippophagy*, or the utilization of horse meat as food. The arguments advanced in favor of it are almost unanswerable. Nothing but disuse, prejudice, or mere sentimentality can be brought against it, or the possession, which we do not have, of a sufficiency of beef, mutton, poultry, fish, etc., as to be procurable by all, even the poorest. Hundreds of colts are yearly raised by farmers and breeders, which at maturity are unfit for almost all kinds of work, that, when four or six months old, will make excellent horse-veal, better far than bob-veal and old cow. Hundreds of horses can be daily seen that are much better suited for the shambles than for work, which, after a few weeks' suitable feeding, would be worth from $40 to $60 for meat. The labors of Mr. Bergh would then be much lightened. In 1878 over 11,000 horses were killed for food, producing over four millions of pounds of flesh. In Berlin at least 5,000 horses are killed yearly for food, and in Altona, about 1,500. Horse meat is regarded by Decroix, an enthusiastic hippophagist, as more healthy and nutritious than that of the ox, sheep, or pig. No other kind of meat should be kept in the shops where horse-beef is sold, and all the animals and meat should be examined by inspectors.

"Glanders" in the horse and man is the only equine disease treated of; and is spread over a long and instructive chapter, reaching from page 159 to 208. The obscurer forms of chronic glanders are especially interesting; see page 165 to 169. In one large stable 36 cases were discovered on the first inspection, and weekly visits among the 1,200 horses, spread over nine months, finally detected 300 diseased animals, all of which were killed, at a loss of $38,000. In another stable nearly 50 were picked out in course of two weeks. So that promptitude is required about it, and also persistence, to stamp it out.

It will have been noticed that almost all the animal diseases treated of in this learned volume are communicable to man, viz.: notably Trichinosis, Tuberculosis, Anthrax, Texas Fever, Hydrophobia, and Glanders. The Doctor does not yet believe in the inter-conveyance of scarlet or typhoid fevers, measles, or cerebro-spinal meningitis, but the mysterious way in which Texas Cattle Fever is carried about, and the large amount of testimony which has accumulated on these points, may well make all hesitate in rejecting it too soon or too positively.

We hope and believe that the volume will be received by all, except perhaps by those especially attacked, with the great welcome that its author and publishers must expect for it. It will take its stand alongside of the popular treatises of Hilliard and Robertson, and on all purely scientific matters will lead them. Either of these works, together with Dr. Billings', will make almost a complete library on Veterinary medicine. J. C. P.

THE INFLUENCE OF HEREDITY AND CONTAGION ON THE PROPAGATION OF TUBERCULOSIS AND THE PREVENTION OF INJURIOUS EFFECTS FROM CONSUMPTION OF THE FLESH AND MILK OF TUBERCULOUS ANIMALS. By Herr A. Lydtin, G. Fleming, LL.D., F.R.C.V.S., and Mr. Van Hersten. London: Bailliere, Tindall and Cox. New York: W. R. Jenkins.

This work is a translation of the report presented at the recent International Veterinary Congress, held at Brussels. At the meeting it was resolved that the disease in question should be included in the Contagious Diseases Act. We promise our readers a more extended review of the work in the July number.

VETERINARY MEDICINE AND SURGERY IN DISEASES AND INJURIES OF THE HORSE. Compiled from Standard and Modern Authorities, and edited by F. O. Kirby. Illustrated by four colored plates and 168 wood engravings. Pp. 332. William Wood & Company, New York, 1883.

This handsome volume comes with no flourish of trumpets, except that inseparate from the imprint of its enterprising publishers. Its author does not even claim to be a great veterinary physician or surgeon, and has prepared it solely for the use of practitioners of medicine and other intelligent horse owners. Fitzwygram's treatise is the basis, and the works of Percivall, Gamgee, Youatt, Mayhew, Williams, Robertson, and many others, the aids, together with his own sixteen years' study and experience in the ownership and care of horses, as the check and guide upon all this good matter.

With all this modesty he does not hesitate, however, to express his belief that this compilation will be found better fitted for its purpose than any similar work which has heretofore been published. And he is right. Every purchaser, scarcely excluding professors and professional veterinarians, will get the full value of his money, and be glad he parted with it.

By the aid of medical men, veterinary medicine has become almost a specialty in medical art and science, and certainly should rank above some of the minor specialties like dentistry and skin diseases. It might well take its place alongside of surgery and the old-fashioned general practitioner.

A reviewer is expected to find some fault, if he can, and accordingly we have pounced upon a few not very important typographical errors, and have found some of the plates not very distinct, or very correctly lettered or numbered; but that is all. The text is fully up to the demands of modern times and accomplished medical men, and will prove a safe guide to all, and easily put not very well posted readers upon an extremely good and even scientific platform.

Science and humanity go hand in hand in this excellent manual, and the absence of all the barbarian superstitions of old horse-masters is refreshing.

The explanation of Plate 1 might well have been put opposite to it, before the title-page; and the table of doses in the very front of the book, in place of the back. All this will show how hard put to it the reviewer was to find any fault. Again, he does not mention what the normal rate of pulse and breathing and temperature of the horse is.

The prescriptions are exceedingly judicious, and the treatment almost if not quite as good as in the same diseases in human beings. He has wisely concluded that the flesh, blood, bones, ligaments, muscles, eyes, lungs, liver, kidneys, and many other parts and organs of the horse, are very similar to those of man, and subject to analogous diseases, so that physicians will easily understand the book.

THE AMERICAN JOURNAL OF OPHTHALMOLOGY. Edited by Adolf Alt, M.D., St. Louis, Mo.

Hitherto ophthalmologists have enjoyed but limited facilities for the publication of the results of their researches and experiences, and we are happy to know that this want will soon cease to be felt, by the appearance of the above named periodical, which is to be published monthly. To bring it within the reach of all, the price of the journal will be only $2.50 a year. We hope to see the efforts of the publishers crowned with success.

THE DOG. By Dr. Al Watts. Boston, 1884.

This little book, of 50 pages, contains much useful information relating to the breeding and training of dogs, their diseases and treatment.

